# Study of the cement implantation syndrome: A review

**DOI:** 10.1097/MD.0000000000038624

**Published:** 2024-06-14

**Authors:** Yunze Yang, Xianyu Meng, Yiran Huang

**Affiliations:** a Orthopedics and Traumatology, The First Affiliated Hospital of Heilongjiang University of Chinese Medicine, Heilongjiang University of Chinese Medicine, Harbin, Heilongjiang, China; b Heilongjiang University of Chinese Medicine, Harbin, Heilongjiang, China.

**Keywords:** bone cement, bone cement implantation, bone cement implantation syndrome, complications of bone cement implantation surgery

## Abstract

Bone cement implantation syndrome (BCIS) is a critical and potentially life-threatening condition that manifests during implantation. Characterized by a constellation of symptoms, including hypoxemia, hypotension, cardiac arrhythmias, elevated pulmonary vascular resistance, and occasionally cardiac arrest, BCIS typically ensues shortly after cement introduction, albeit with rare instances of delayed onset. Primarily attributed to the exothermic reaction of bone cement implantation, this syndrome is caused by local tissue damage, histamine and prostaglandin release, and microemboli formation, ultimately triggering a systemic immune response that culminates in respiratory and circulatory failure. The current hypotheses regarding BCIS include embolism, allergic reactions, and cement autotoxicity. BCIS management emphasizes preventative strategies, encompassing meticulous patient risk assessment, comprehensive preoperative and intraoperative evaluations, and precise cement application techniques. Treatment primarily involves symptomatic therapy and life-support measures to address the systemic effects of the syndrome.

## 1. Introduction

Bone cement is an orthopedic medical material whose main component is polymethylmethacrylate (PMMA). Bone cement has excellent biocompatibility and physical properties and can play an important role in procedures such as fracture repair and artificial joint replacement. By injecting bone cement, the fracture site can be strengthened and stabilized, promoting bone healing and restoring bone function. In addition, bone cement can be used to fill bone defects, correct bone structure, and fix and implant artificial joints. First used in total hip replacement in 1951. Bone cement implantation syndrome (BCIS) involves a series of pathophysiological disorders of the respiratory and circulatory systems that occur during bone cement implantation surgery. The main clinical manifestations are hypoxemia, hypotension, cardiac arrhythmias, increased pulmonary vascular resistance (PVR), and cardiac arrest.^[1]^ These clinical signs may be associated with embolisms, allergic reactions, and material toxicity. With improvements in medical materials and advances in medicine, the incidence of BCIS has decreased, but the fatality rate of severe BCIS remains high.^[2]^

## 2. Epidemiological characteristics of BCIS

Kiaer first applied bone cement after total hip arthroplasty in 1951, and Powell^[3]^ reported circulatory failure after implantation of bone cement in the first femoral head replacement in 1970. This phenomenon has occurred frequently during implantation of bone cement, which was formally named BCIS by Byrick.^[4]^ BCIS is most commonly observed during hip and knee arthroplasties. It occurs in the following 4 stages: femoral reaming, cement placement, prosthesis placement, joint repositioning, and rarely, during tourniquet deflation.

The incidence of BCIS ranges from 0.14% to 0.68% for total hip arthroplasty and 0.4% to 4.3% for hemiarthroplasty, with mortality rates ranging from 0.09% to 0.29%.^[5]^ BCIS is the most important cause of intraoperative and postoperative death and disability following cemented artificial hip arthroplasty. BCIS is the most important cause of intraoperative and postoperative death and disability in cemented artificial hip replacement. There is a huge statistical discrepancy between the incidence of BCIS and the mortality rate, mainly due to the lack of uniformity in diagnostic methods and the lack of awareness among physicians, as well as the fact that some of the early studies have confused BCIS with pulmonary embolism.^[6]^ Therefore, it is of great practical significance to enhance doctors’ awareness of BICS and correct use of bone cement, and to formulate specifications for the clinical use of bone cement in order to guarantee the safety of patients’ lives.^[7]^

## 3. The pathogenesis of BCIS

The main component of bone cement is PMMA, which causes high temperatures and pressures in the medulla during implantation. The entry of bone cement, fat particles, and air emboli into the bloodstream results in complex physiological processes. Kotyra et al reported that after bone cement and prosthesis implantation, mean arterial pressure (−10%), cardiac index (−10%), and per-pulse output index (−10%) decreased, whereas PAPs (10%–15%) and pulmonary vascular resistance index (45%) were elevated.^[8]^ Pulmonary hemodynamics and right ventricular variables changed progressively over time, whereas the intrapulmonary shunt and physiological dead space increased immediately after prosthesis implantation and then returned to baseline levels. Segerstad et al demonstrated a 45% and 20% increase in the pulmonary vascular resistance index (PVRI) during and after prosthesis implantation in the cemented and non-cemented groups, respectively (*P* < .005). Systolic and mean arterial pressures increased by 18% and 17%, respectively, in the cemented group but not in the uncemented group (*P* < .001).^[9]^ There was a trend toward a more pronounced decrease in right ventricular ejection fraction in the cemented group, whereas there was no difference in cardiac output or output per beat between the 2 groups. The implantation of bone cement has been shown to increase PVRI and right ventricular afterload, but the pathogenesis of BCIS remains unclear. The main hypotheses are embolic, allergic reaction, and PMMA toxicity hypotheses.

### 3.1. Embolization hypothesis

The embolization doctrine is the main doctrine of the BCIS. The presence of vascular and pulmonary intravascular emboli has been confirmed by esophageal ultrasound observations, animal experiments, and clinical results.^[10,11]^ According to Guo trial, inferior vena cava filters are effective in preventing BCIS, and as a corollary, pulmonary embolism is the main cause of BCIS symptoms.^[12]^ However, most microemboli dislodged from bone cement are small in diameter and only obstruct microvessels. Therefore, whether the dislodged microemboli stimulate the coagulation system leading to thrombus aggregation or whether the activation of inflammatory mediators leading to vascular inflammatory lesions leading to thrombus aggregation should be considered. According to Mordà et al, despite the fact that surgery leads to a shift in the layout of the thromboela-stogram toward hypercoagulability, this is similar in cemented and uncemented surgical interventions.^[13]^ It was concluded that coagulation changes were not a cause or factor in determining BCIS, but the mean age of the study 81.91 years may have had some limitations across the age range. Most BCIS occurs within 3 minutes (mucous filament phase), which is significantly different from the time of occurrence of pulmonary embolism.^[2,14]^ The embolization theory is difficult to convince all researchers, but right ventricular failure secondary to elevated pulmonary arterial pressure is thought to be the primary cause of systemic hypotension and cardiac arrest.^[15]^

### 3.2. Anaphylaxis hypothesis

The clinical symptoms of BCIS are similar to those of anaphylactic reactions and include decreased blood pressure, increased heart rate, and decreased body temperature. Lamadé hypothesized that the release of histamine into the bloodstream from the body tissues caused by tiny particles of bone cement, leading to an allergic reaction, causes the pathogenesis of BCIS; however, the use of H1-receptor antagonists does not reduce the pathogenesis of BCIS.^[16]^ Anaphylaxis occurred in a clinical case at Ten Hagen, but histamine receptor blockers have no prophylactic potential in BCIS, and the patient had underlying systemic indolent mastocytosis, a rare pathological disorder that may amplify an allergic reaction after massive systemic mast cell activation.^[17]^ A study^[18]^ reported modest protection upon blocking histamine receptors with clemastine and cimetidine (H1 and H2 antagonists); however, these findings failed to be replicated in subsequent investigations.^[16,19]^ Based on current evidence, while histamine may play a role in the pathogenesis of BCIS, it remains inconclusive whether it is the sole cause of this condition.

### 3.3. The PMMA toxicity hypothesis

PMMA has significant circulatory and coagulation toxicity, including monotoxic reactions to the bone cement itself, such as effects on cardiac muscle, endothelial cells, and ion channels. It also includes a large number of thermal reactions that occur during the cement setting. The thermal reaction acts on the surface of bone cells and leads to local tissue necrosis, and the large amount of necrotic tissue together with the released histamine and prostaglandins further deteriorates circulatory and coagulation system disorders. However, the toxicity of PMMA is related to its physicochemical properties and blood delivery, and studies have shown that the amount of PMMA used is not sufficiently toxic to cause BCIS.^[18]^

The embolism theory is limited by the transient nature of certain BCIS transients, thereby reducing its explanatory power.^[14]^ Similarly, the anaphylactic reaction theory is hindered by the inability of histamine antagonists to prevent BCIS development^[15]^ and the absence of mast cell activation during autopsy,^[20]^ questioning its validity. Furthermore, the concentration of bone cement monoliths in the bloodstream is insufficient to induce cardiovascular symptoms, further limiting the plausibility of this theory.^[21]^ Collectively, these observations underscore the need for a more comprehensive understanding of BCIS etiology.

In essence, none of the aforementioned hypotheses fully account for the etiology of BCIS or encapsulate its clinical manifestations, which rather emerge as a harmonious blend of these proposed mechanisms.^[15]^

## 4. Diagnosis of BCIS

There is no standardized diagnosis for BCIS, and its early manifestations include a decrease in systemic blood pressure, an increase in pulmonary vascular pressure, a decrease in right ventricular ejection fraction, and a decrease in cardiac output accompanied by laboratory markers of platelet lowering. Vigilance and close detection should be performed during bone cement application, as severe diseases most often progress by the time circulatory or consciousness disturbances occur. It should be differentiated from pulmonary and fat embolism. The use of transesophageal echocardiography aids in the early detection of BICS.^[22]^

Although BCIS occurs mostly within 50 seconds-3 minutes of implantation of cement implantation, in a few patients, BCIS occurs within a few hours postoperatively.^[23]^ Delayed onset BCIS is not easily detected in time, and is usually detected in severe cases.

The BCIS grading system uses Donaldson criteria for 3 levels, based on oxygen saturation.^[1]^

Grade 1: oxygen saturation (SPO2) < 94% or arterial systolic blood pressure (SBP) decreased by > 20%.

Grade 2: SPO2 < 88%, SBP > 40% lower than usual, or sudden loss of consciousness.

Grade 3: cardiac arrest or respiratory failure requiring cardiopulmonary resuscitation.

## 5. Prevention of BCIS

The prevention of BCIS is critical, and once it occurs, it can cause irreversible damage to the patient. Preoperative meticulous assessment and relentless intraoperative vigilance are paramount in the prevention and management of BCIS, particularly in the absence of consensus on the optimal anesthetic technique for cemented bone surgery within the scientific literature.^[24]^

### 5.1. Patient risk assessment

Patients should be evaluated for a high prevalence of BCIS, such as those with cardiovascular disease, congenital patent foramen ovale, age > 75 years, American Society of Anesthesiologist score III to IV, malignant neoplastic disease, bone diseases such as osteoporosis, comorbid cardiopulmonary disease, and co-infections.^[25–28]^ Performing a patient risk assessment helps physicians be alert to the occurrence of BCIS by pre-planning preoperatively, testing vital signs intraoperatively, and monitoring the patient for several hours postoperatively.

### 5.2. Preoperative assessment

Relevant laboratory and physical examinations, blood and urine routine, coagulation, liver and kidney function, etc, ultrasound test, dynamic electrocardiogram, etc, should be perfected before the operation. Special attention should be paid to the comprehensive assessment of the patient general state and attempts to achieve the ideal state before surgery. During surgery, intravenous access should be established in a timely manner to expand the volume, reduce plasma viscosity, increase effective perfusion rate, accelerate blood circulation, and reduce PMMA tissue toxicity. However, guided hemodynamic therapy is ineffective in reducing the incidence of BCIS.^[29]^

### 5.3. Intraoperative monitoring

The choice of intraoperative anesthesia also affects the incidence of BCIS, with simple epidural anesthesia increasing the incidence of BCIS compared with spinal epidural anesthesia.^[30]^ In addition, the patient heart rate, blood pressure, blood oxygen level, and other indicators should be closely monitored. The amount of infusion should be adjusted according to the changes in blood pressure during surgery, and vasoactive substances should be used to assist in raising blood pressure if necessary. If the patient bleeds heavily during surgery, an appropriate blood transfusion can be used to maintain normal blood levels and avoid tissue ischemia and hypoxic injury. Maintaining the stability of intraoperative anesthesia and physiological indices is a key factor in avoiding BCIS.

### 5.4. Precautions for bone cement implantation

Avoiding simultaneous bilateral implantation of cemented prostheses, choosing the prosthesis with the smallest range of surgical requirements, using low-viscosity cements, and avoiding the use of cements in high-risk patients. Lowering the pressure in the bone marrow cavity while implanting bone cement is key to reducing the occurrence of BCIS. Intraoperative pressure in the bone marrow cavity can be reduced by vacuum mixing of the bone cement, flushing of the bone marrow cavity, and thorough removal of residual bone marrow tissue.^[31]^ Intraoperatively, a drain can be placed into the medullary cavity prior to cement placement and removed at the time of cement placement to expel the air remaining in the medullary cavity, thereby effectively reducing intramedullary pressure.^[7]^

## 6. Treatment of BCIS

Different levels of therapeutic measures are implemented according to the degree of the patient condition, and patients with serious conditions should be admitted to the ICU for resuscitation treatment. Changes in vital signs should be closely monitored and symptomatic treatment should be administered.

Rapid fluid replacement to maintain the patient right heart preload while taking care that the volume of rehydration should not be excessive to prevent lowering of the left cardiac output and falling into heart failure. Fluid replacement should be accompanied by attention to vital signs, based on hemodynamics, and appropriate use of vasoactive drugs to ensure adequate myocardial perfusion and coronary artery oxygen demand to maintain normal cardiac function and correct cardiac arrhythmias, such as αβ-agonists. Zcan supplementation with colloids prior to surgery to avoid intravascular hypovolemia has played a role in preventing BCIS.^[32]^

Therefore, oxygen therapy should be administered to these patients. When BCIS occurs, the patient will have symptoms of hypoxia, and oxygen therapy should be administered to the patient promptly. Depending on the patient symptoms, different types of oxygen therapy should be administered to increase the arterial partial pressure of oxygen. Oxygen therapy should be administered when the partial pressure is stabilized at 94% or above to prevent sudden and dangerous conditions. If conventional oxygen therapy fails to effectively elevate the patient partial pressure of oxygen, assisted ventilation measures such as tracheal intubation and tracheotomy should be implemented to ensure the correction of hypoxia and prevent irreversible damage to multiple organs due to hypoxia.

The patient coagulation function was monitored and the patient plasma fibrinogen level was measured to avoid thrombosis and exacerbation of BCIS due to excessive coagulation. Heparin can be administered to patients as anticoagulant therapy if necessary.

Drug therapy, which can effectively reduce the inflammatory response and tissue damage in the occurrence of BCIS, smoothening hemodynamics, inducing the formation of anti-inflammatory factors, inhibiting the synthesis of inflammatory factors, and inducing shock, is administered to patients. In addition, antihistamines are used to reduce the release of histamine from necrotic tissue, which causes anaphylaxis.^[16]^

In cases of low SBP, rehydration should be accelerated and ephedrine and dopamine should be administered intravenously. Norepinephrine and epinephrine should also be used to prevent cardiac arrest.^[22]^ Patients with a clear central thrombus should undergo prompt surgery to remove the thrombus to avoid interfering with blood transport, thus affecting tissue oxygenation.

It has been theorized that one of the reasons for pulmonary vasoconstriction in BCIS is serotonin. Nevertheless, as a precautionary measure, ondansetron, a specific antagonist of the 5-hydroxytryptamine receptor, can be used both before and during surgery.^[33]^

## 7. Bone cement development

PMMA bone cement has undergone many iterations and advancements since its inception, and has been characterized by its own characteristics. Primary bone cements use highly viscous hand-mixed bone cements, which are pressed into the bone marrow cavity by acupressure, are not adequately rinsed out of the bone marrow cavity, and the bone cements are not adequately bonded, and do not have a high degree of purity or surface smoothness. Step by step, a bone cement with lower viscosity is used for better fluidity, vacuum mixing is performed to eliminate air bubbles in the interstitial space of the bone cement, the bone marrow cavity is flushed with high pressure to completely remove the residual blood clots and bone fragments in the bone marrow cavity, and the bone cement is injected with a pressurized gun to make it completely integrated with the bone of the prosthesis. The probability of loosening of the implanted prosthesis was reduced. Simultaneously, reduction in tissue damage and better tissue fusion effectively reduced the incidence of BCIS.

Moreover, PMMA bone cement has more bio-additives to meet different needs and minimize the complications of bone cement implantation, such as bioceramic additives, filler additives, antimicrobial additives, porogens, biologics, and mixing additives.^[34]^

## 8. Conclusion

BCIS is a serious orthopedic surgical complication, giving patients and healthcare workers great risks and injuries, the pathogenesis of which is not completely clear. The treatment is not perfect; it is the main symptomatic treatment, providing patients with respiratory and circulatory support. Recognizing the risk of BICS, understanding the high-risk groups for BICS, fully recognizing the symptoms of BICS occurrence, and treating patients with BICS in a timely and symptomatic manner is key to reducing the mortality rate. With the development of science and technology, as well as a clearer understanding of the pathogenesis of BICS, BICS will definitely be completely conquered.

## Author contributions

**Conceptualization:** Yunze Yang.

**Formal analysis:** Yunze Yang.

**Investigation:** Yunze Yang.

**Methodology:** Yunze Yang.

**Supervision:** Yunze Yang, Xianyu Meng, Yiran Huang.

**Validation:** Yunze Yang, Xianyu Meng, Yiran Huang.

**Writing – original draft:** Yunze Yang, Yiran Huang.

**Writing – review & editing:** Yunze Yang, Xianyu Meng.
